# Dietary Approaches for Japanese Patients with Diabetes: A Systematic Review

**DOI:** 10.3390/nu10081080

**Published:** 2018-08-13

**Authors:** Satoru Yamada, Yusuke Kabeya, Hiroshi Noto

**Affiliations:** 1 Diabetes Center, Kitasato Institute Hospital, 5-9-1 Shirokane, Minato-ku, Tokyo 108-8642, Japan; 2 Department of Home Care Medicine, Saiyu Clinic, 3-217-1 Sagamicho, Koshigaya-shi, Saitama 343-0823, Japan; ykabeyan@yahoo.co.jp; 3 Endocrinology Department, St. Luke’s International Hospital, 9-1 Akashicho, Chuo-ku, Tokyo 104-8560, Japan; noto-tky@umin.net

**Keywords:** energy restricted diet, low energy diet, carbohydrate restricted diet, low carbohydrate diet, diabetes, Japanese

## Abstract

This study aimed to elucidate the effect of an energy restricted and carbohydrate restricted diet on the management of Japanese diabetes patients. Several databases including MEDLINE, EMBASE, and the Japan Medical Abstracts Society were searched for relevant articles published prior to June 2017. The articles identified were systematically reviewed. We identified 286 articles on an energy restricted diet, assessed seven and included two studies in our review. On a carbohydrate restricted diet, 75 articles were extracted, seven articles assessed and three included in the review, of which two were the studies that were selected for the energy restricted diet group, since they compared energy restricted diets with carbohydrate restricted diets. All selected studies were on Japanese patients with type 2 diabetes. No studies for type 1 diabetes were found in our search. Two randomized controlled trials on an energy restricted diet were also included in the three studies for a carbohydrate restricted diet. All the three randomized controlled trials showed better glucose management with the carbohydrate restricted diet. Our study revealed that there is very little evidence on diets, particularly in Japanese patients with diabetes, and that the energy restricted diet, which has been recommended by the Japan Diabetes Society in the sole dietary management approach, is not supported by any scientific evidence. Our findings suggest that the carbohydrate restricted diet, but not the energy restricted diet, might have short term benefits for the management of diabetes in Japanese patients. However, since our analysis was based on a limited number of small randomized controlled trials, large scale and/or long term trials examining the dietary approaches in these patients are needed to confirm our findings.

## 1. Introduction

Nutrition therapy plays an integral role in the management of diabetes [1]. Several dietary approaches such as the Mediterranean diet, dietary approaches to stop hypertension (DASH), a vegetarian diet, and a carbohydrate restricted diet have been recommended by the American Diabetes Association (ADA) and reviewed by Ley et al. in *The Lancet* [2,3]. On the other hand, the Japan Diabetes Society (JDS) has officially recommended only an energy restricted diet since 1965 [4], as it believes that the pathophysiology and food preferences of Japanese patients with diabetes are unique when compared to patients in Western countries [5]. As per the JDS guidelines, energy restriction was calculated based on the ideal body weight as follows: total energy intake (kcal) = ideal body weight (kg = height (m) × height (m) × 22) × physical activity index (obese, 20–25; non-obese sedentary, 25–30; non-obese normal intensity active, 30–35; non-obese high intensity active, 35 and above). In fact, a recent study of the trajectory of body mass index (BMI) in Japanese patients with diabetes has shown it to be continuously normal (approximately 24.4) and not obese [6]. The BMI of Japanese individuals is very different from that of Americans [7] and Europeans [8], suggesting that there must be differences in the pathophysiology as well. However, because the European Association for the Study of Diabetes (EASD) mentioned that energy restriction is not needed in patients whose BMI is lower than 25 [9], the discrepancy in the position of energy restriction between the JDS and the Western countries seems more problematic. Although the JDS guidelines [4] on the dietary recommendations are reportedly based on 86 studies, none of those references support the JDS recommended an energy restriction diet. Eighty-three of these papers were not studies on dietary approach for Japanese patients with type 2 diabetes. The remaining three references were on dietary approaches for Japanese diabetic patients, where one is the ‘eating vegetables before carbohydrate’ diet [10], one is the before-after study on exercise and energy restriction diet which instructed a deficit of 140 kcal/d from the baseline for subjects with metabolic syndrome [11], and the last is the carbohydrate restriction diet [12]. Thus, it is evident that the JDS guidelines are not based on any supportive evidence for energy restriction in diabetic patients with normal BMI, despite the fact that energy restriction is the basis of the consensus in preparing the guidelines by the writing committee [4]. However, since the JDS recognizes that an evaluation of the effectiveness and safety of dietary approaches is needed in their recommendation [5], in this study we have tried to evaluate the effectiveness of the energy restricted diet in Japanese patients with diabetes. Furthermore, in recent years, emerging evidence has suggested that a restricted carbohydrate diet improves glycemic control [13], although it is not yet conclusive, especially in long term follow-ups [1,14]. We have, therefore, evaluated the effectiveness of the carbohydrate restricted diet as well.

## 2. Materials and Methods

### 2.1. Study Search

Searches of MEDLINE, EMBASE, and Japan Medical Abstracts Society (JAMAS) databases from their inception (MEDLINE 1966, EMBASE 1947, AND JAMAS 1964) until 30 June 2017, were performed. To identify studies related to an energy restricted diet, we used a combination of the following keywords: “low-energy” or “energy-restriction” or “low-calorie” or “caloric-restriction” and “diabetes,” and “Japanese” in the MEDLINE and EMBASE databases. Similarly, for articles related to a carbohydrate restricted diet, we used the terms “low-carbohydrate” or “carbohydrate-restriction,” and “diabetes,” and “Japanese” in the MEDLINE and EMBASE databases. In the JAMAS database, we used the same combination of keywords in Japanese. Other dietary approaches such as, the Mediterranean diet, DASH (dietary approaches to stop hypertension), fat restriction (low fat) diet, low glycemic index (low GI) diet, vegetarian diet, and high-protein diet were excluded because preliminary searches by one author (S.Y.) found no relevant study in Japanese (partly presented in 60th JDS annual meeting in 2017, Nagoya by S.Y.).

### 2.2. Study Selection

The following exclusion criteria were applied: (1) non-Japanese data, (2) non-diabetic patient data, (3) other dietary approaches, (4) unpublished data (including abstracts presented only in scientific meetings), and (5) studies not appropriate for our evaluation such as case series and case reports.

After each search, based on the title and abstract, two authors (S.Y. and Y.K.) extracted relevant reports independently. One author (S.Y.) collected the papers for a full-text evaluation by two independent authors (S.Y and Y.K). Disagreements were primarily resolved through discussions and by consulting a third author (H.N.). We did not perform any quantitative data analysis because our search found few studies that were not enough to perform a meta-analysis.

### 2.3. Validity and Quality Assessment

The risk of bias was assessed against the key criteria: random sequence generation; allocation concealment; blinding of participants, personnel, and assessors; incomplete outcome data; selective outcomes reporting; and other sources of bias, in accordance with the recommendations of the Medical Information Network Distribution Service (Minds) [15].

### 2.4. Data Abstraction

We reviewed each full-text report to determine its eligibility and extracted and tabulated all the relevant data independently. The extracted data included the characteristics of the subjects (including age, gender), the study design, publication year, follow-up period, and risk parameters. Any disagreement was resolved by consensus among the investigators.

## 3. Results

**Figure 1:**
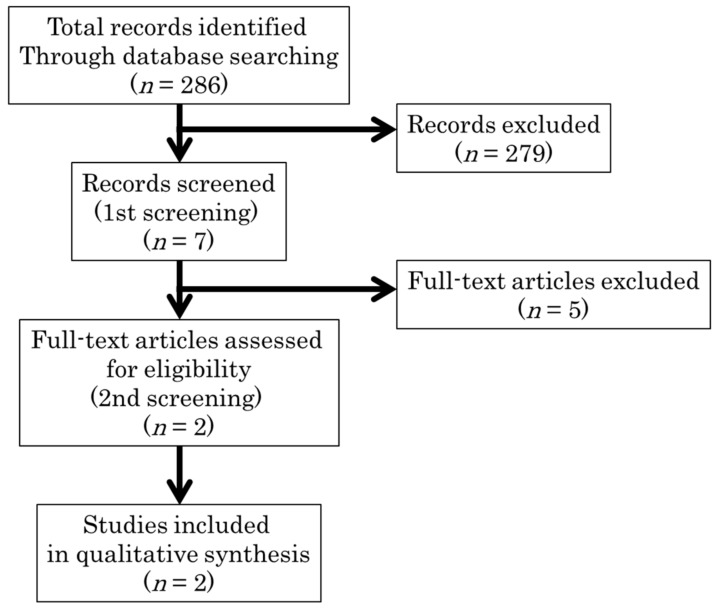
Flow diagram of selecting the energy restriction studies included in our study. Two randomized controlled trials were appraised in our systematic review.

We identified a total of 286 articles related to an energy restricted diet, of which seven [16,17,18,19,20,21,22] were assessed for their eligibility for inclusion in our review (Figure 1). All were studies on patients with type 2 diabetes. No study was identified for type 1 diabetes. There were no articles outlining the adverse effects of energy restriction. Five articles were excluded from the systematic review because (a) counseling [16], (b) meal delivery [17], and (c) periodization [18] were evaluated under the same level of energy restriction in three studies; one study assessed the effects of very strict energy restriction under hospitalization (1000 kcal/day) [19]; while another did not evaluate an energy restricted diet [20]. After excluding these five studies, the remaining two randomized controlled trials (RCTs) were appraised in our systematic review [21,22]. The two selected articles were moderately homogeneous in terms of the level of energy restriction. Both studies adopted a carbohydrate restricted diet as the control group. The sample sizes in these two studies were 24 [21] and 66 [22].

Both studies were also selected in the carbohydrate restricted diet section. To avoid redundancy, we created a summary table that included the two studies each on a carbohydrate restricted diet and an energy restricted diet, and an energy restricted diet was used as the control group (Table 1). In both studies, the energy restricted diet (Table 1, control group) had inferior effects on HbA1c improvement when compared to the carbohydrate restriction group (Table 1, intervention group). However, a review of dietary energy in one study [21] revealed that the levels of energy intake were similar in both the energy and carbohydrate restriction groups. In the other study [22], the levels of energy intake were higher in the energy restriction group compared to those in the carbohydrate restriction group. Thus, we concluded that the net effect of energy restriction on glycemic control could not be assessed in both studies (Table 2).

**Figure 2:**
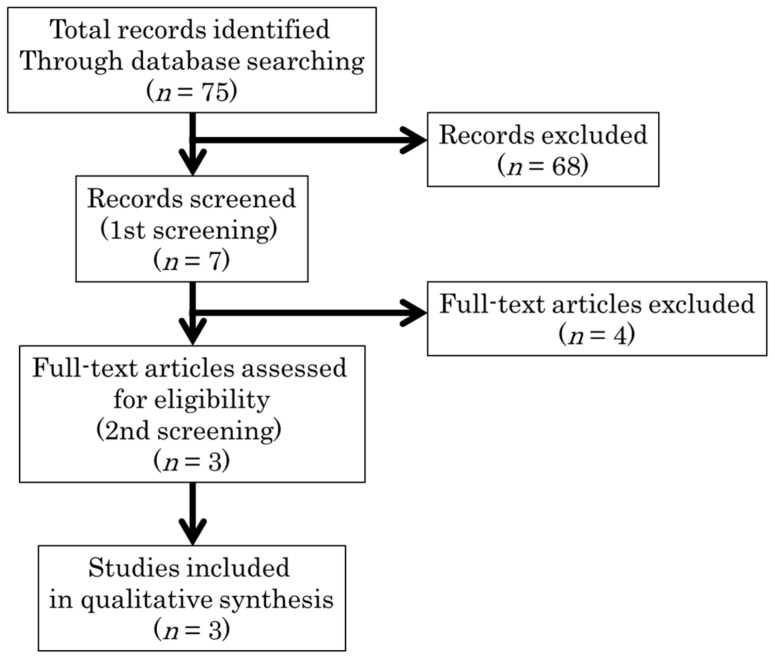
Flow diagram of selecting the carbohydrate studies included in our study. Three randomized controlled trials were appraised in our systematic review.

We identified a total of 75 articles related to a carbohydrate restricted diet, of which seven were assessed for their eligibility to be included in our review (Figure 2) [19,20,21,22,23,24,25]. All were studies on patients with type 2 diabetes. No studies were identified for type 1 diabetes. There were no articles that showed adverse effects of carbohydrate restriction. Four articles were excluded from the study because the same levels of carbohydrate restriction were recommended for all participants, and a longitudinal change in glycemic control was observed in two studies [20,23]. One study evaluated the effect of very strict energy restriction under hospitalization (1000 kcal/day) [19]. In one study, carbohydrate restriction was prescribed only on a single day (admission day 8) [24]. After excluding these four studies, the remaining three RCTs were appraised in our systematic review [21,22,25]. The three selected articles were moderately homogeneous in terms of the level of carbohydrate restriction. Among these studies, two had an energy restricted diet as the control groups and were the same studies selected for the energy restricted diet analysis [21,22], while the third study had a carbohydrate-rich diet with similar energy intake [25]. The sample sizes in these three studies were 24 [21], 66 [22], and 15 [25].

Table 1 shows the summary of each study. A carbohydrate restricted diet had superior effects on improvement of HbA1c [21,22] or the postprandial glucose levels as determined by continuous glucose monitoring [25] compared to the control group in all three studies. However, in one study the energy intake was lower in the intervention (carbohydrate restriction) group than in the control (energy restriction) group [22] (Table 2). Thus, we concluded that a carbohydrate restriction diet was supported by limited evidence in Japanese patients with type 2 diabetes.

## 4. Discussion

The present study was an attempt to elucidate the effects of an energy restricted diet and carbohydrate restricted diet on the management of diabetes in Japanese patients by reviewing previous studies from available databases. The first and most important finding of our study is that there is very little evidence on diets in Japanese patients with diabetes.

Our second finding was that an energy restricted diet, which has been recommended by the JDS as the sole dietary management approach, is not supported by any scientific evidence so far. The two studies in our review were not appropriate to judge the effects of an energy restricted diet on glycemic control because the levels of energy intake in the energy restriction group were not lower than those in the control group [21,22]. While in one study the energy restriction group showed a non-significant reduction of HbA1c (from 7.7 ± 0.6 to 7.5 ± 1.0%, *p* = 0.45) [18], no change was seen in HbA1c in another study [22], indicating that an energy restricted diet is not supported by scientific evidence in Japan. The available data on the effects of an energy restricted diet are very limited, which results in difficulties in the evaluation and recommendation of such a diet. Furthermore, the CALERIE (Comprehensive Assessment of Long term Effects of Reducing Intake of Energy) study revealed that energy restriction in non-obese subjects leads to loss of bone mineral density [26] and lean body mass [27] in a two year period. In light of these findings, a multi-faceted evaluation might be required for patients with diabetes in terms of examining the effects and safety of energy restriction. If the JDS continues to adopt dietary guidelines [4] different from those in Western countries, it should be backed by sufficient evidence that can be critically evaluated. Well-designed, multi-centered RCTs to establish dietary evidence should be actively encouraged.

The third finding of our study was that as a short-term management approach a carbohydrate restricted diet is more effective than an energy restricted diet. All three RCTs in our review were small but well-designed [21,22,25]. Although observational studies have presented safety concerns regarding the carbohydrate restricted diet in Western countries [28,29], a similar study in Japan was in favor of carbohydrate restriction [30]. Furthermore, recently the PURE (Prospective Urban and Rural Epidemiological) study, which was held in 18 countries, showed that carbohydrate intake was positively correlated with mortality [31], suggesting the safety of carbohydrate restriction.

Regarding the long-term effects of carbohydrate restriction, Sato et al. have recently reported that the statistical superiority of a carbohydrate restricted diet receded in an 18-month follow-up study. In their cohort, the median HbA1c level changed from 8.3% at baseline to 8.2% at 18 months in the energy restriction group and from 8.0% to 7.7% in the carbohydrate restriction group [32]. Hence, Sato et al. concluded that well-constructed nutrition therapy, including both energy and carbohydrate restricted diets, might be equally effective in improving HbA1c levels in the long term [32]. However, improvement in HbA1c have not yet been observed with energy restricted diets. Furthermore, although it was a non-RCT, Haimoto et al. [33] have previously shown that the carbohydrate restriction group had better HbA1c, BMI, and tapering of sulfonylureas during a 24-month period. Sanada et al. [34] have reported that a carbohydrate restriction diet was effective during a 36-month period. Thus, we cannot exclude the possibility that a carbohydrate restricted diet is effective in the long term. Unlike other carbohydrate restriction studies, the study by Sato et al. [22,32], showed that the carbohydrate restriction group consumed less energy than the energy restriction group. This reduced energy intake in the energy non-restricted group may explain the rebound in the carbohydrate restriction group in the study by Sato et al. [32]. Our findings therefore point towards the possibility that a carbohydrate restricted diet could potentially be an option as a first line dietary approach for Japanese patients with diabetes. However, longer-term and larger RCTs are needed to confirm this hypothesis.

Our study has some limitations. First, all three studies included in our review were small and short term. Second, the included studies all have a potential performance bias because the subjects and healthcare providers were not blinded. However, it might be difficult to solve this problem in studies evaluating dietary effects on glycemic control under actual clinical situations.

## 5. Conclusions

Our systematic review revealed that there are only a few dietary studies in Japanese patients with diabetes. Large-scale trials are needed to more fully evaluate the risks and benefits of an energy restricted diet, which is recommended by JDS. On the other hand, the effectiveness of a carbohydrate restricted diet is supported by limited evidence, at least in the short term. Well-designed and more sophisticated trials are needed to establish evidence-based dietary approaches specifically for these patients.

## Figures and Tables

**Table 1:** Summary table of studies on carbohydrate restriction diet (including energy restriction studies as control group [21,22]).

Study ID (Reference Number)	Setting	Study Design	Patients					Intervention	Control	Primary Outcome	Results		
			Number of Participants	Number of Dropouts	Age	Sex (M/F)	Duration of Diabetes (Years)				Intervention	Control	Median Difference
Yamada et al., 2014 [21]	Outpatient University Hospital	RCT	24	C: 0 E: 0	C: 63.3 ± 13.5 E: 63.2 ± 10.2	C: 7/5 E: 5/7	C: 8.9 ± 3.6 E: 9.5 ± 4.8	Carbohydrate: 70–130 g/day	IBW (kg) ×25 kcal/day	HbA1c change after 6 month	−0.6 ± 0.48	−0.2 ± 0.68	−0.40
Sato et al., 2017 [22]	Outpatient University Hospital	RCT	66	C: 1 E: 3	C: 60.5 ± 10.5 E: 58.4 ± 10.0	C: 23/7 E: 24/8	(median) C: 14.0 E: 13.0	Carbohydrate 130 g/day	IBW (kg) x 28 kcal/day	HbA1c change after 6 month	(median)-0.65	(median) 0.00	−0.65
Yabe et al., 2017 [25]	Meal test 2 Medical Institutions	RCT	15	C: 0 H: 0	C: 56.9 ± 7.3 H: 54.3 ± 5.2	C: 7/0 E: 5/3	C: 7.6 ± 4.3 E: 4.4 ± 3.3	Carbohydrate 180 g/day, Energy 1800 kcal/d	Carbohydrate 247.5 g/day, Energy 1800 kcal/day	CGM data during 5−7 days	130.32 ± 27.72	142.92 ± 39.6	n.a.

C: carbohydrate restriction group = intervention group, E: energy restriction group = control group [21,22], H: high carbohydrate group=control group, RCT: randomized controlled trials. M = male, F = female. IBW: ideal body weight. n.a.; not available.

**Table 2:** Methodological quality and risk of bias of carbohydrate restriction studies (including energy restriction studies as control group [21,22]).

Study ID [Reference Number]	Sample Size Calculation	Risk of Bias								Indirectness				
		Random Sequence Generation	Allocation Concealment *	Blinding of Participants and Personnel *	Blinding of Outcome Assessment *	Incomplete Outcome Data	Selective Reporting	Other Source of Bias	Study free from total biases *	Subjects	Intervention	Control	Outcome	Total Indirectness
Yamada et al., 2014 [21]	Yes	Yes	Low	Unclear	Unclear	Low	Low	Unclear	Unclear	No	No	No	No	No
Sato et al., 2017 [22]	Yes	Yes	Low	Unclear	Unclear	Low	Low	Unclear	Unclear	Yes **	Yes ***	No	No	No
Yabe et al., 2017 [25]	n.a.	Yes	Low	Unclear	Unclear	Low	Low	Unclear	Unclear	Yes **	Yes ****	Yes ****	No	Yes

* In dietary study, blinding and concealment are impossible. Thus, no dietary study is unbiased. ** In these studies [22,25], female/male ratio was different between intervention and control group. *** In this study [22], energy intake is strictly restricted to the intervention group. **** This study [25] used test meals supplied by Nichirei Foods Inc. (Tokyo, Japan).
